# No antagonism or cross-resistance and a high barrier to the emergence of resistance *in vitro* for the combination of islatravir and lenacapavir

**DOI:** 10.1128/aac.00334-24

**Published:** 2024-06-12

**Authors:** Tracy L. Diamond, Shih Lin Goh, Winnie Ngo, Silveria Rodriguez, Min Xu, Daniel J. Klein, Jay A. Grobler, Ernest Asante-Appiah

**Affiliations:** 1 Merck & Co., Inc., Rahway, New Jersey, USA; IrsiCaixa Institut de Recerca de la Sida, Barcelona, Spain

**Keywords:** human immunodeficiency virus, HIV, islatravir, lenacapavir, antiretroviral resistance, *in vitro*

## Abstract

Islatravir (ISL) is a deoxyadenosine analog that inhibits HIV-1 reverse transcription by multiple mechanisms. Lenacapavir (LEN) is a novel capsid inhibitor that inhibits HIV-1 at multiple stages throughout the viral life cycle. ISL and LEN are being investigated as once-weekly combination oral therapy for the treatment of HIV-1. Here, we characterized ISL and LEN *in vitro* to assess combinatorial antiviral activity, cytotoxicity, and the potential for interactions between the two compounds. Bliss analysis revealed ISL with LEN demonstrated additive inhibition of HIV-1 replication, with no evidence of antagonism across the range of concentrations tested. ISL exhibited potent antiviral activity against variants encoding known LEN resistance-associated mutations (RAMs) with or without the presence of M184V, an ISL RAM in reverse transcriptase (RT) . Static resistance selection experiments were conducted with ISL and LEN alone and in combination, initiating with either wild-type virus or virus containing the M184I RAM in RT to further assess their barrier to the emergence of resistance. The combination of ISL with LEN more effectively suppressed viral breakthrough at lower multiples of the compounds’ IC_50_ (half-maximal inhibitory concentration) values and fewer mutations emerged with the combination compared to either compound on its own. The known pathways for development of resistance with ISL and LEN were not altered, and no novel single mutations emerged that substantially reduced susceptibility to either compound. The lack of antagonism and cross-resistance between ISL and LEN support the ongoing evaluation of the combination for treatment of HIV-1.

## INTRODUCTION

Islatravir (ISL) and lenacapavir (LEN) are antiretroviral inhibitors currently in development as a once-weekly oral combination treatment regimen for HIV-1. These compounds block HIV-1 replication with different mechanisms of action, act at different stages of the viral life cycle, and have been demonstrated to be effective *in vitro* and *in vivo*.

ISL is a deoxyadenosine analog that is converted intracellularly to its pharmacologically active triphosphate form (ISL-TP) via endogenous cellular kinases. ISL-TP is a potent nucleoside reverse transcriptase translocation inhibitor (NRTTI) that inhibits HIV-1 reverse transcriptase (RT) by multiple mechanisms (1–4). ISL-TP is different from approved nucleoside reverse transcriptase inhibitors (NRTIs) in that it maintains a 3′-hydroxyl and is thus not an obligate chain terminator. Upon incorporation of ISL, the 4′-ethynyl group of the molecule typically causes immediate chain termination by inhibiting translocation. If translocation does occur, the 3′-hydroxyl group can be utilized to add one more nucleotide, but further elongation is blocked due to RT either being misaligned for further extension or disassociating from the primer:template. ISL-TP can also be misincorporated (opposite nucleobases G, C, or A, rather than its cognate substrate nucleobase T or U), leading to immediate chain termination when the mismatched primer is unable to be extended and/or excised.

LEN is a novel capsid (CA) inhibitor that inhibits multiple stages of the virus life cycle (5). LEN directly binds to the interface between capsid protein subunits. It is a potent inhibitor of early stages of the HIV-1 replication cycle, where it is hypothesized to compete directly with host nuclear import proteins binding to CA, preventing nuclear import of preintegration complexes. LEN also blocks virus assembly and release (late stage), where it interferes with Gag/Gag-Pol function, reducing production of intracellular Gag and processed CA. Additionally, LEN accelerates the rate of capsid subunit association, resulting in virions with malformed CA cores.

Decreases in susceptibility to ISL and LEN have previously been associated with mutations in the genes that encode HIV-1 RT and CA, respectively, and therefore, the two compounds are not expected to have overlapping resistance profiles (6–9). Thus, the combination of ISL and LEN has the potential to be a highly effective treatment option for HIV-1.

Described here are several *in vitro* studies which support the combination of ISL and LEN for the treatment of HIV-1 infection. Combination assays were performed to determine if the compounds acted synergistically, additively, or antagonistically for antiviral activity. Although not expected, a potential to exert enhanced cytotoxic effects on cells was also assessed. The activity of ISL was also investigated against variants containing LEN resistance-associated mutations (RAMs) with and without M184V RT [one of the most common ISL RAMs selected *in vitro* (6, 10–12)]. In addition, the barrier to the emergence of resistance of the independent compounds and the combination were assessed *in vitro* in selection experiments starting with wild-type (WT) or M184I RT encoding virus. The emergent viruses were characterized and did not reveal additional single codon variants that altered the susceptibility to either ISL or LEN.

## MATERIALS AND METHODS

### Multiple-cycle antiviral assay in MT4 cells which express green fluorescent protein upon infection (MT4-GFP cells)

ISL and LEN were evaluated for their ability to prevent HIV-1 infection in MT4-GFP cells as described in Diamond et al. (6). In this method, HIV-1 replication is monitored using MT4-gag-GFP clone D3, MT4 cells with a GFP reporter gene, the expression of which is dependent on the HIV-1 expressed proteins, Tat and Rev (13). Infection of MT4-GFP cells with HIV-1 results in GFP expression approximately 24 h post-infection. Proviral variants used for these studies were generated by site-directed mutagenesis in the WT R8 proviral plasmid (14). Virus was produced by transfection in 293T cells.

Briefly, MT4-GFP cells were cultured overnight with the appropriate HIV-1 variant at a multiplicity of infection (MOI) that resulted in approximately 100 green (infected) cells at 48 h post-infection in the wells containing no inhibitor. This was done to ensure similar assay signal-to-noise ratios between experiments and to enable work with viral variants demonstrating a range of viral fitness properties. After ~19 h of infection, the cells were washed and resuspended in assay media containing normal human serum (NHS) before their addition to 384-well compound plates containing 10-point serial 3-fold dilutions of ISL or LEN. Controls included no inhibitor [dimethyl sulfoxide (DMSO) only] and a combination of three antiviral agents, efavirenz, indinavir, and the integrase strand transfer inhibitor, L-002254051, at final concentrations of 4 µM each. The infected/treated cells were cultured at 37°C, 5% carbon dioxide, and 90% humidity until evaluation of infection was conducted at approximately 48 and 72 h post-infection by counting the number of green fluorescent cells in each well using an Acumen ^e^X3 scanner (TTP Labtech Inc., Melbourn, UK). The reproductive ratio, *R*, was calculated by dividing the number of infected cells at 72 h by the number at 48 h post-infection. The percent inhibition caused by a test compound is calculated by the following formula:

% inhibition = [1 − (*R*_test compound_ – *R*_positive control_)] / (*R*_DMSO only_ – *R*_positive control_) × 100%.

The dose-response curves of each test compound were plotted as percent inhibition versus the test concentration. IC_50_ values were determined by a non-linear four˗parameter curve fitting of the dose-response curve data. Fold-change compared to WT was used to assess antiviral activity across compounds and variants.

### Antiviral activity of the combination of ISL with LEN

The antiviral assay described above was utilized to test the antiviral activity of the combination of ISL with LEN. Using WT R8 (subtype B) virus infection of MT4-GFP cells, a matrix of ISL and LEN concentrations was tested, with each cross-titration performed in quadruplicate. Briefly, ISL was evaluated using a 12.81-nM high-test concentration with eight additional dilutions (concentration range: 0.50–12.81 nM). Each dilution of ISL was tested in combination with six dilutions of LEN (concentration range = 0.021–5.0 nM). Each compound was also tested on its own. As a positive control for an antagonistic combination, stavudine (d4T, concentration range: 10 nM to 3 µM), was combined with ribavirin (RBV, concentration range: 4.1 nM to 27.0 µM). Cytotoxicity was also assessed after 24-h incubation on a separate plate with uninfected MT4-GFP cells using the CellTiter-Glo assay (Promega). Analysis of the combined antiviral activities and cytotoxicity was conducted using the MacSynergy II three-dimensional model for statistical evaluation of drug-drug interactions (15).

### Static resistance selection method for ISL/LEN combinations

*In vitro* selection experiments were performed in 96-well plates using R8 (subtype B) WT virus or R8 virus encoding M184I RT, with ISL and LEN alone or in combination at concentrations defined by fixed multiples over each compound’s IC_50_ against R8 WT virus, previously determined in the multiple-cycle antiviral assay in MT4-GFP cells with 10% NHS (ISL IC_50_ = 0.79 nM, LEN IC_50_ = 0.087 nM). Compound plates were prepared with 1 µL of each compound at 200× the final concentration (2 µL total) and stored at −80°C. One 96-well plate was used for each compound alone or the combination. Different inhibitor concentrations (indicated as fold multiples of WT IC_50_) were studied across the columns (see Fig. 3A and 4A; Table S1) . Rows A–H represent eight independent replicates of the concentrations studied in columns 1–12.

For cell culture, MT4-GFP cells were infected in bulk with R8 WT virus or R8 virus encoding M184I RT at a high MOI of 5. Seventy thousand infected cells were added per well to a room temperature compound plate for a final volume of 200 µL per well. Plates were incubated for 4  days at 37°C, 5% carbon dioxide, and 90% humidity prior to initiation of the next selection passage. The supernatant was passaged to a new compound plate with uninfected MT4-GFP cells every 3–4  days, and the compound concentrations were kept constant throughout the experiment. The number of GFP-positive cells in each well was monitored at each passage using an Acumen eX3 scanner (TTP Labtech Inc.). Viral breakthrough was defined as a well with >100 GFP-positive cells. Using this criterion, the number of wells with breakthrough stabilized by passage 6; six additional passages were performed before genotyping was conducted.

Analysis of CA or RT mutation(s) in the breakthrough viruses from the resistance selection studies was performed using population-based Sanger sequencing at passage 12. Viral RNA was extracted with the MagMAX 96 viral RNA isolation kit from the culture supernatant of wells with viral breakthrough from the resistance studies described above. The CA or RT-encoding region was amplified by the one-step RT-PCR method. PCR products were genotyped by an automated population-based full-length sequencing method (covering amino acids 1–231 of the CA region and 1–440 of the RT region). The primers used for PCR amplification of CA were 5′-CAACCATCCCTTCAGACAGG-3′ (forward) and 5′-GCTGTTGGCTCTGGTCTGCTC-3′ (reverse). The sequencing primers were 5′-CCAAGGAAGCCTTAGATAA- 3′, 5′-GTGCATGCAGGGCCTATTGC-3′, 5′-GCTGGTAGGGCTATACATTC-3′, and 5′-CTTTTTCCTAGGGGCCCTG-3′. The primers used for PCR amplification of RT were 5′-AAGCAGGAGCCGATAGACAA-3′ (forward) and 5′-TAATCCCGAATCCTGCAAAGCT-3′ (reverse). The sequencing primers were 5′-CCCTGTGGAAGCAC-3′, 5′- GGATGTGGGCGATGC-3′, 5′-TTGACTCAGATTGGCTGCAC-3′, and 5′-TCCTCTGTCAGTTACATATCCT-3′. Sequencing results were reported as amino acid changes compared with R8 (subtype B) (accession no. KT200357.1) or R8 encoding M184I RT (RT codon 184 mutated by site-directed mutagenesis from ATG→ATA).

## RESULTS

### ISL with LEN demonstrated additive inhibition of HIV-1 replication with no evidence of antagonism

**Fig 1:**
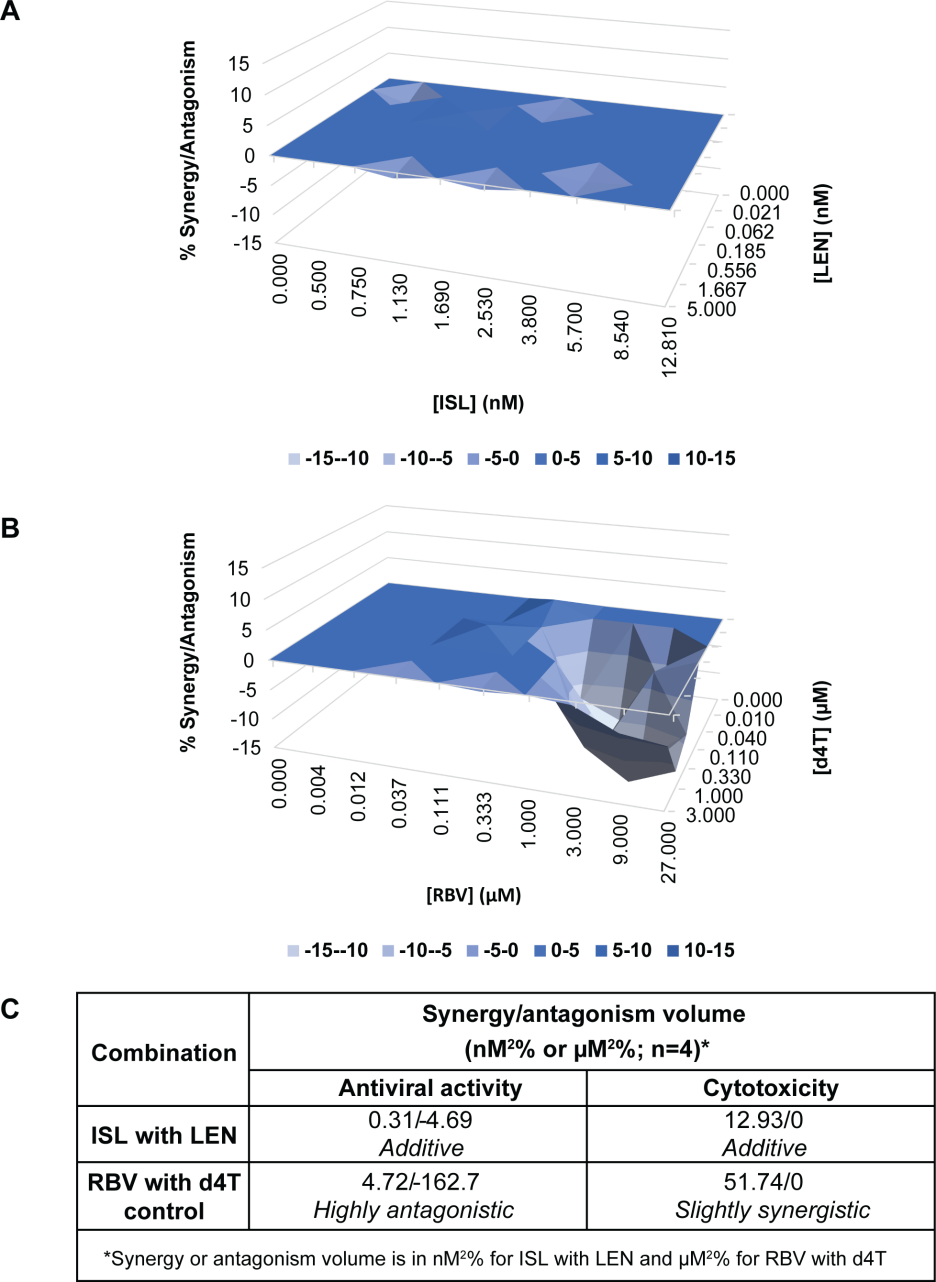
Combination antiviral activity and cytotoxicity results. Antiviral assays for the combination of ISL with LEN (**A**) or RBV with d4T (**B**) were performed using the multiple-cycle antiviral assay in MT4-GFP cells infected with WT HIV-1 (R8) virus. Results were analyzed using the MacSynergy II three-dimensional model for statistical evaluation of drug-drug interactions (15) to assess whether the interactions were significantly different from additive (i.e., synergistic or antagonistic). (**C**) Synergy/antagonism volumes are shown as 95% confidence values for the combinations using the multiple-cycle antiviral assay or a cytotoxicity assay in MT4-GFP cells and can be interpreted as follows: additive, 50 to −50; slightly synergistic, >50 to 100; highly synergistic, >100; slightly antagonistic, <−50 to 100; highly antagonistic, <−100.

To assess potential antagonistic interactions between ISL and LEN that could affect the ability of the combination to inhibit HIV-1 replication, the antiviral activities of the combination across a range of concentrations were evaluated in a multiple-cycle antiviral assay using the combination of d4T and RBV as a positive control for antagonism (16). Combinations of ISL and LEN had an additive effect on the inhibition of HIV-1 replication, with synergy and antagonism volumes of 0.31 and −4.69 nM^2^ %, respectively (Fig. 1A and C). In contrast, the control pair, d4T and RBV, was highly antagonistic in inhibiting HIV-1 replication, with an antagonism volume of −162.7 µM^2^ % (Fig. 1B and C).

To assess whether the combination of ISL and LEN could result in enhanced cytotoxicity, ATP levels from uninfected MT4-GFP incubated with the compounds were also measured via a cytotoxicity assay. The combination of ISL with LEN did not show synergistic cytotoxicity (was additive), with a synergy volume of 12.93 nM^2^ % (Fig. 1C), whereas the combination of d4T with RBV was slightly synergistic with respect to cytotoxicity, with a synergy volume of 51.74 µM^2^ % (Fig. 1C).

Altogether, *in vitro* ISL and LEN combination studies revealed no evidence of antagonism of the two drugs, with respect to inhibition of HIV-1 replication, and no notable enhanced cytotoxicity in MT4-GFP cells.

### ISL exhibited potent (nM) antiviral activity against published LEN resistance-associated variants with or without M184V-containing RT, and LEN antiviral activity was not affected by the addition of RT M184V

**Fig 2:**
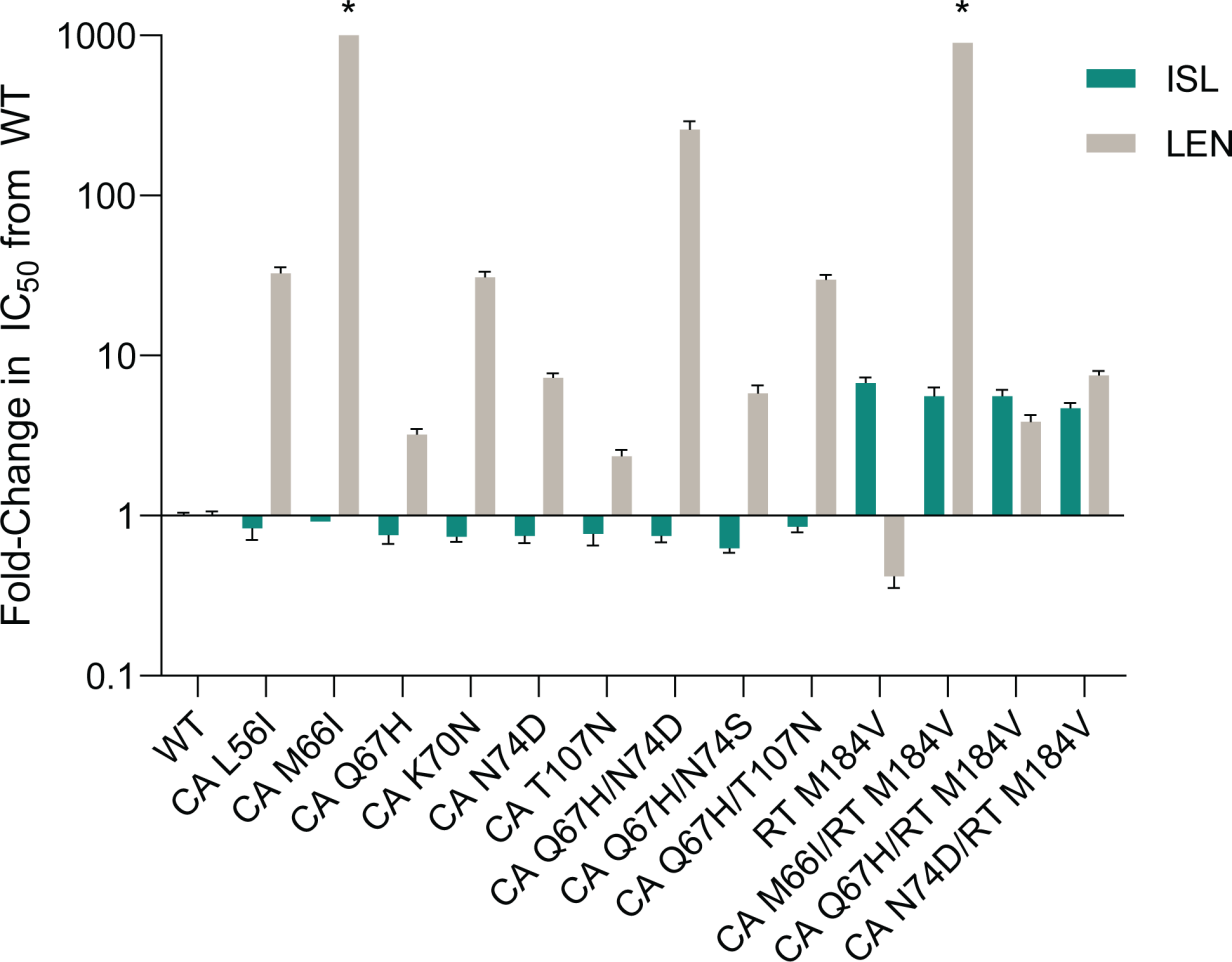
Antiviral activity of ISL and LEN against viruses encoding published LEN RAMs compared to WT R8 virus in 100% NHS. The mean (+SEM) is shown for the fold-change in the IC_50_ of ISL (teal) or LEN (gray) of each variant compared to WT. *Fold-changes for CA M66I and CA M66I/RT M184V were >1,758.0- and >917.3-fold, respectively.

LEN RAMs in the region encoding CA have been previously reported from *in vitro* resistance selection studies (5). The impact of these CA substitutions on susceptibility to ISL or LEN was evaluated in parallel using the MT4-GFP multiple-cycle antiviral assay in 100% NHS. The antiviral activities of ISL and LEN against 9 LEN resistance-associated variants were compared to that against the WT R8 virus; the fold-change in IC_50_ of each variant compared to WT R8 is shown for each compound (Fig. 2; Table S2). ISL maintained potent activity against all nine LEN resistance-associated variants, with IC_50_ values ranging from 0.65 to 0.96 nM, comparable to that against the WT virus. As expected (5, 17, 18), LEN demonstrated reduced antiviral activity against all of the LEN resistance-associated variants. Notably, L56I, M66I, K70N, Q67H/N74D, and Q67H/T107N variants resulted in more than 10-fold reduced susceptibility to LEN compared to WT R8.

As mutations in the region encoding RT position 184 are the most common mutations associated with resistance to ISL (6, 7, 10–12, 19), the effects of M184V mutation combined with LEN RAMs on susceptibility to LEN and ISL were also examined in the MT4-GFP antiviral assay. M184V alone resulted in decreased susceptibility to ISL (fold-change = 6.8) and slightly increased susceptibility to LEN (fold-change = 0.4) (Fig. 2; Table S2). The addition of M184V to M66I, Q67H, or N74D CA mutations did not result in further decreased susceptibility to ISL (fold-change = 4.8–5.7; Fig. 2; Table S2). Similarly, the antiviral activity of LEN against variants with M184V, in combination with LEN RAMs, was similar to the activity against the variants without M184V (Fig. 2; Table S2).

### The combination of ISL with LEN more effectively suppressed viral breakthrough and demonstrated a higher barrier to the emergence of resistance than either compound on its own

**Fig 3:**
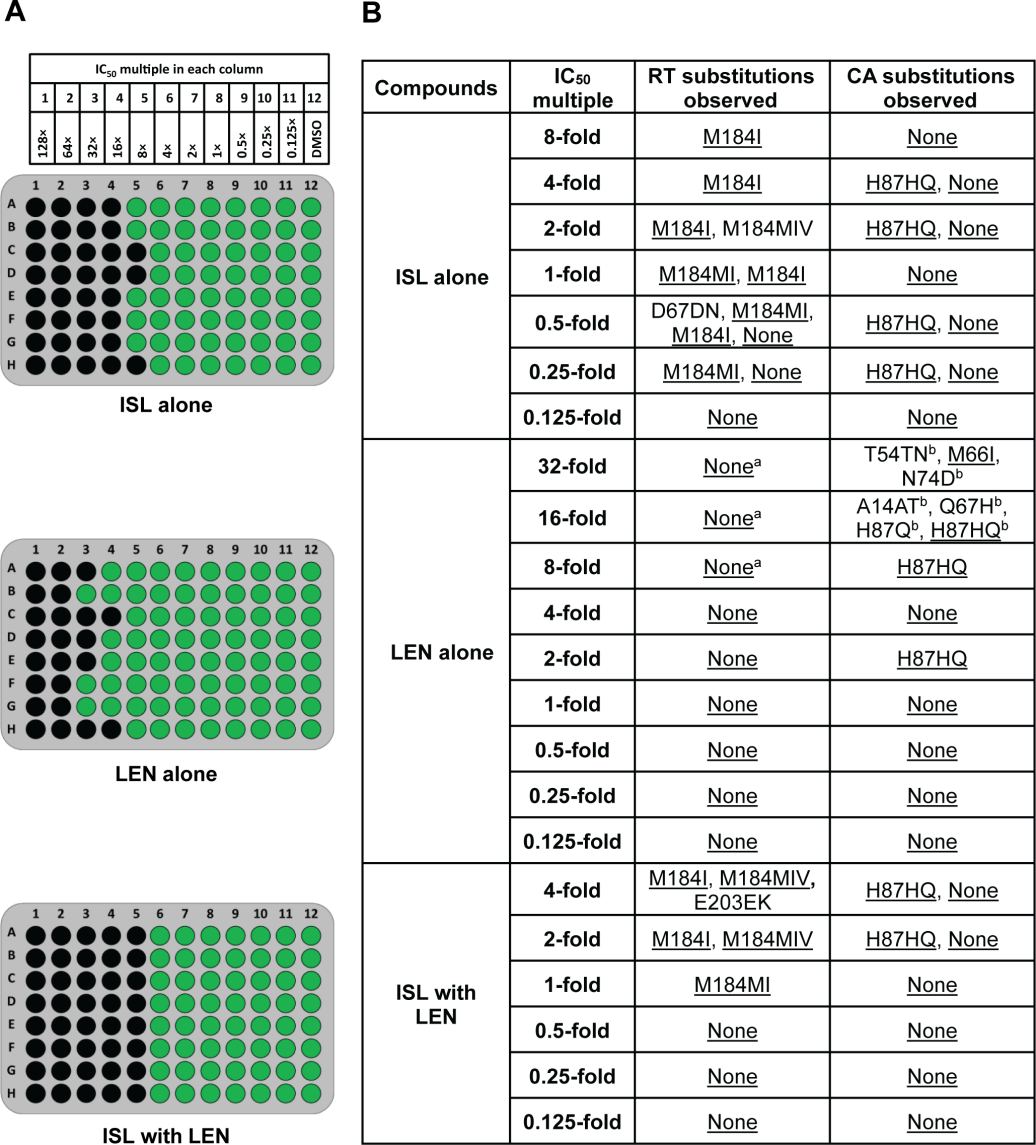
Static resistance selection experiment in MT4-GFP cells with WT R8 virus. Images of plates are shown, with green indicating wells with viral breakthrough (containing >100 GFP-expressing cells) and black wells indicating cells without viral breakthrough (**A**). Mutations encoding RT and CA substitutions observed in resistance selection experiments at each compound’s IC_50_ multiple are also shown (**B**). Substitutions are depicted by the single-letter amino acid (AA) code within the reference sequence followed by the AA position number and then the single-letter AA codes predicted by the sequencing analysis. Multiple AA codes are shown if a mixture of AA codes for the same position was observed in a single well. “None” indicates that no changes from the WT R8 starting virus were observed. Underlined substitutions represent those seen in more than one well across all IC_50_ multiples on each plate. ^a^E28K, R277K, M357T, and N418S RT substitutions were observed in a few wells in the LEN alone selection experiment in the same wells as M66I or Q67H CA substitutions and are not considered relevant to LEN. N418S RT substitution has been previously observed without selective pressure with the same virus stock.^b^T54TN was observed with N74D in a single well. A14AT was observed in a single well with H87HQ. Q67H was observed in a single well with H87Q.

**Fig 4:**
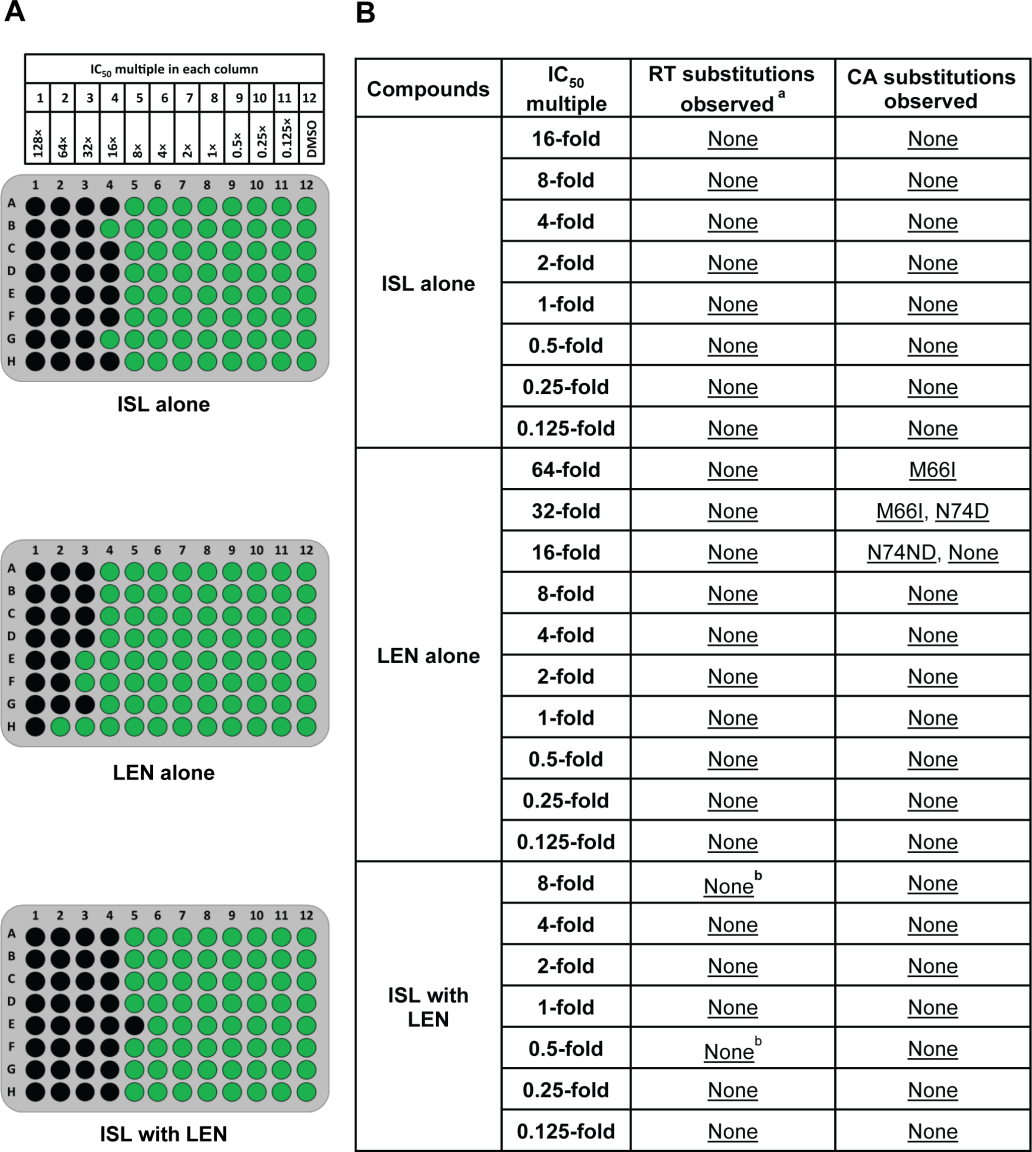
Static resistance selection experiment in MT4-GFP cells initiated with RT M184I (R8) virus. Images of plates are shown, with green indicating wells with viral breakthrough (containing >100 GFP-expressing cells) and black wells indicating cells without viral breakthrough (**A**). Mutations encoding RT and CA substitutions observed in resistance selection experiments at each IC_50_ multiple are also shown as compared to the RT M184I starting virus (**B**). Substitutions are depicted by the single-letter AA code within the reference sequence followed by the AA position number and then the single-letter AA codes predicted by the sequencing analysis. Multiple AA codes are shown if a mixture of AA codes for the same position was observed in a single well. “None” indicates that no changes from the RT M184I starting virus were observed. Underlined substitutions represent those seen in more than one well across all IC_50_ multiples on each plate. ^a^There was no reversion of M184I observed. ^b^T386TI substitution was observed in a single well each at 8.0- and 0.5-fold of each compound’s IC_50_ in the ISL with LEN selection experiment and is considered a polymorphism.

To assess whether the combination of ISL with LEN could more effectively suppress viral breakthrough than either compound on its own, static resistance selection experiments were conducted *in vitro*. MT4-GFP cells infected with WT R8 virus were treated with the combination of ISL and LEN at multiples of each compound’s IC_50_ against WT R8 virus (see Fig. 3A and 4A; Table S1). Parallel selection experiments were also conducted with ISL or LEN alone.

For individual compound selections, ISL suppressed viral breakthrough in all wells with concentration of >8-fold of its IC_50_, while LEN suppressed viral breakthrough in most wells with >16-fold of its IC_50_ (Fig. 3A). The combination of ISL with LEN effectively suppressed breakthrough in all wells with compound concentrations of >4-fold of either compound’s IC_50_. Therefore, the combination was more effective at suppressing viral breakthrough, at 2-fold lower multiples compared to ISL alone and at 4-fold lower multiples compared to LEN alone.

Genotypic analysis was performed on wells that demonstrated virus breakthrough (Fig. 3B). In the ISL-only, LEN-only, and combination of ISL with LEN selection experiments, H87Q CA mutations were observed as a mixture with wild-type H87 in multiple wells (H87Q without wild-type only occurred in a single well). H87Q was not observed in wells without selective pressure (with DMSO instead of compound). Over 90% of isolates with CA sequence available in the Stanford University HIV Drug Resistance Database have Q87 rather than the consensus H87 (20), indicating that this mutation is likely a polymorphism and is not related to resistance.

In ISL-only selections, M184 RT mutations, typically M184I or M184MI (a mixture of wild type and M184I detected in the same well), were observed in all wells with viral breakthrough at an IC_50_ multiple of ≥1-fold. In LEN-only selection experiments, mutations encoding amino acid changes in CA other than H87Q were observed in 5 out of 10 wells with viral breakthrough at 16- to 32-fold of the LEN IC_50_. M66I was detected in two wells at 32-fold of the LEN IC_50_. In addition to M66I, LEN-only selection also resulted in the following CA mutations that were only observed in single occurrences at ≥16-fold the LEN IC_50_: A14AT, T54N, N74D, and Q67H. A14T and T54N CA substitutions have not been described previously. In selections combining ISL with LEN starting with the WT virus, M184 RT-encoding mutations were observed in all wells with breakthrough virus at or above each compound’s IC_50_. No CA mutations besides H87HQ were observed in the combination selection experiment.

Since M184I was detected in most wells with breakthrough virus selected against the combination of ISL with LEN, separate resistance selection experiments were conducted, starting with an M184I RT-containing HIV-1 in MT4-GFP cells (Fig. 4). ISL alone suppressed breakthrough in most wells with >8-fold of the ISL IC_50_ for WT virus, while LEN suppressed breakthrough in most wells with >16-fold of its IC_50_ (Fig. 4A). The combination of ISL with LEN effectively suppressed viral breakthrough in all wells with >8-fold of each compound’s IC_50_. The results aligned with observations from selection experiments initiated with the WT virus, as described above, and are consistent with the decreased susceptibility of the M184I variant to ISL [fold-change = 6.2 (6)].

Genotypic analysis of wells with virus breakthrough was conducted for the selection experiments starting with M184I RT-encoding virus. Analysis revealed that M184I was maintained in all wells (there was no reversion of M184I to WT) from experiments conducted with each compound alone or with the combination (Fig. 4B). Furthermore, no additional RAMs in the region encoding RT were detected. In experiments with ISL alone, no changes were observed in CA. In experiments with LEN alone, the M66I CA-encoding mutation was observed in one well each at 64- and 32-fold of the LEN IC_50_. Additionally, N74D was observed separately in two wells at 32-fold of the LEN IC_50_, whereas N74ND was observed in three wells at 16-fold of the LEN IC_50_. In the selection experiments with the combination of ISL with LEN, no mutations in the region encoding CA were observed. Therefore, the genetic barrier to the emergence of resistance appears to be higher for the combination compared to LEN alone.

### No novel single mutation substantially impacted the potency of ISL or LEN

**Fig 5:**
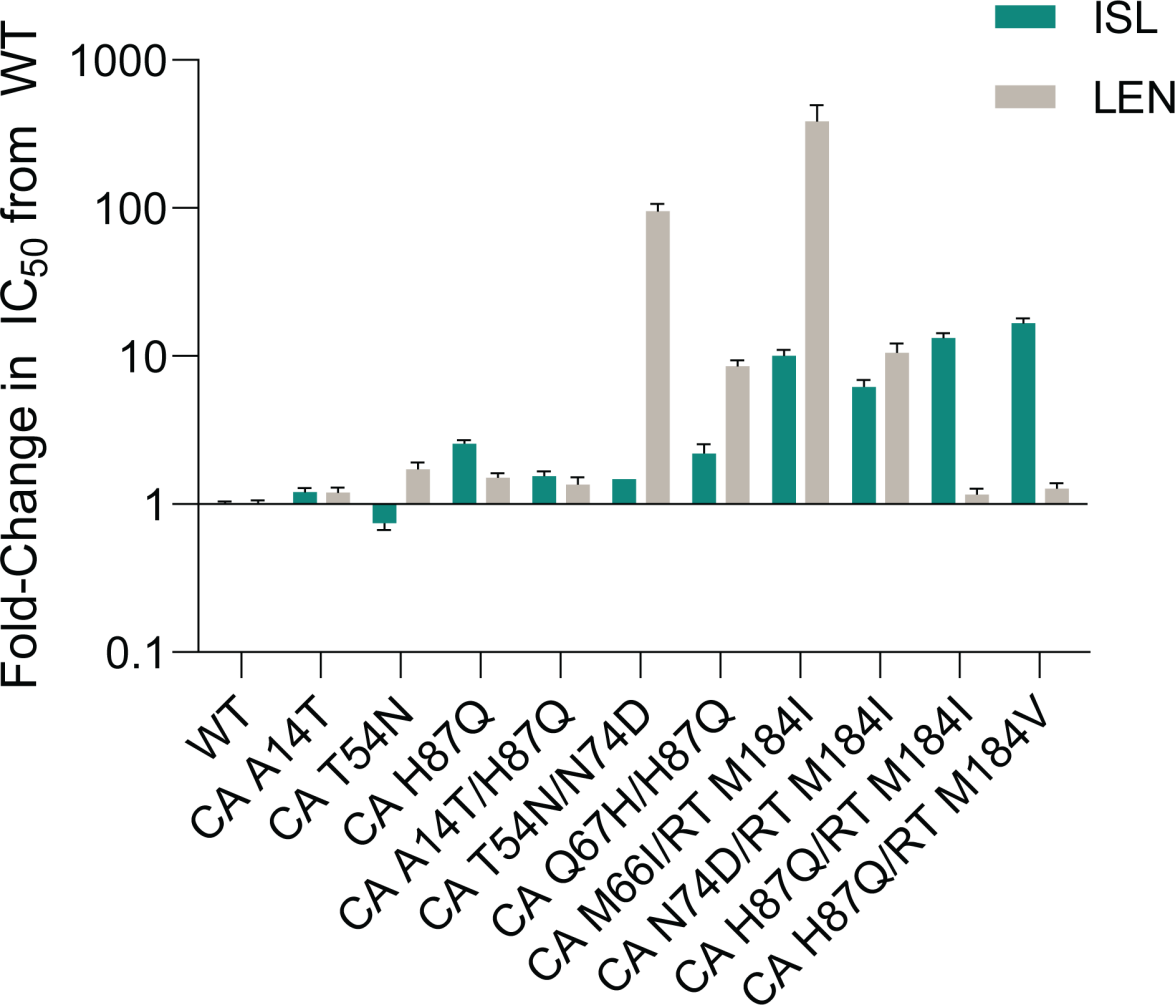
Antiviral activity of ISL and LEN against emergent variants from static resistance selection experiments in 100% NHS. The mean (+SEM) is shown for the fold-change in IC_50_ of ISL (teal) or LEN (gray) against each variant compared to WT.

Since the combination resistance selection experiments identified several novel mutations in the region encoding CA, HIV-1 variants were made to evaluate their susceptibility to ISL or LEN in the multiple-cycle MT4-GFP antiviral assay in 100% NHS (Fig. 5; Table S3). ISL maintained high potency against variants that contained only CA substitutions, with IC_50_ values ranging from 0.77 to 2.77 nM, all within <3-fold of its potency against WT R8. In addition, ISL maintained potency within 3-fold of its potency against M184I or M184V [6.2- or 6.8-fold, respectively (6)] for the variants with a CA substitution combined with M184I or M184V RT substitution (6.3- to 17.1-fold). Variants CA M66I/RT M184I, CA N74D/RT M184I, and CA H87Q/RT M184I demonstrated ISL IC_50_ values between 6.71 and 14.34 nM, similar to that of M184I alone [6.56 nM (6)]. Combining M184V RT and H87Q CA mutations resulted in a slightly larger potency shift, 17-fold less potent compared to WT R8, though still achieving an IC_50_ of 18.1 nM.

LEN maintained high potency against most of the novel CA variants, including variants that combined M184I or M184V RT mutations (Fig. 5; Table S3). Single-substitution variants CA A14T, T54N, and H87Q had similar susceptibility to WT with 1.2- to 1.5-fold decreased susceptibility compared to WT (IC_50_ from 2.92 to 4.21 nM). Combination variants CA A14T/H87Q, CA H87Q/RT M184I, and CA H87Q/RT M184V demonstrated IC_50_ values ranging between 2.84 and 3.32 nM, all within 2-fold of LEN’s potency against WT R8. Variants that contained CA mutations M66I or N74D, in combination with RT M184I, demonstrated reduced susceptibility to LEN similar to that observed with the same CA mutations in combination with RT M184V. Moreover, CA Q67H/H87Q demonstrated reduced susceptibility to LEN similar to that observed with the CA substitution Q67H alone (above, Fig. 2; Table S2). On the other hand, the variant with the combination of CA T54N and N74D demonstrated a 97.5-fold reduced susceptibility to LEN compared to a 7.4- or 1.8-fold reduction in susceptibility for the variants with N74D or T54N alone, respectively, compared to WT R8 (Fig. 2 and 5; Tables S2 and S3).

Altogether, while the combination resistance selection experiments identified several novel CA mutations, none of the novel single mutations substantially impacted susceptibility of the variant to LEN or ISL. A potential interaction between a known LEN RAM encoding CA N74D and a novel mutation encoding CA T54N was identified.

## DISCUSSION

ISL and LEN are in development as a once-weekly oral combination therapy that has the potential to be a highly effective treatment for people living with HIV-1 (PLWH). Multiple studies have previously been conducted to evaluate the antiviral activity and genetic barrier to resistance of the individual molecules *in vitro*, demonstrating high potency against wild-type virus and common resistance-associated variants (6, 8). Here, the activity and resistance patterns for the combination were evaluated *in vitro*.

First, antiviral assays to assess the potential interactions between ISL and LEN were performed. No antagonism between ISL and LEN was observed, and the two-drug combination demonstrated an additive effect on inhibiting virus replication within the concentration ranges tested. Furthermore, the combination did not reveal enhanced cytotoxicity.

LEN RAMs in the CA-encoding region which map to the LEN-binding site have previously been reported (5): variants encoding CA L56I, M66I, Q67H, K70N, N74D, Q67H/N74D, Q67H/N74S, and Q67H/T107N exhibited 6- to >3,200-fold reduction in susceptibility to LEN. Similarly, using the multiple-cycle HIV-1 inhibition assay, these variants conferred 2.4- to >1,758-fold reduction in susceptibility to LEN. Results from each individual variant were mostly within 3-fold of those previously reported for LEN, with L56I, Q67H/N74D, and Q67H/N74D variants having slightly smaller reductions (f4- to 7-fold) than those previously reported. Notably, ISL remained potent against all the CA variants, with no significant fold-changes in IC_50_ (0.6- to 0.9-fold) observed compared to WT virus. The results are consistent with previous studies that showed many of these LEN resistance-associated variants remained susceptible to inhibitors from other antiretroviral classes, such as efavirenz (non-nucleoside reverse transcriptase inhibitor), dolutegravir (integrase strand transfer inhibitor), and tenofovir alafenamide (NRTI) (5, 18).

The antiviral activities of ISL and LEN against variants containing combinations of CA mutations (M66I, Q67H, or N74D) with the RT M184V mutation, a commonly reported mutation in PLWH that is associated with resistance to ISL, also showed an absence of cross-resistance. The results revealed that the addition of M184V did not abrogate sensitivity to LEN, whereby the reductions in susceptibility to LEN were all within 2-fold of that of the single CA variants. The variants also remained susceptible to ISL, conferring an expected 4.8- to 5.7-fold reduction in susceptibility compared to WT virus, comparable to the 6.8-fold reduction in susceptibility previously reported for the variant containing RT M184V alone (6).

As ISL and LEN act at different stages of the viral life cycle through distinct inhibitory mechanisms, the two-drug combination could more effectively suppress viral breakthrough compared to the individual compounds but could also result in the emergence of novel RAMs. *In vitro* resistance selection experiments showed that the combination of ISL with LEN inhibited viral replication and breakthrough in wells at lower compound concentrations than that observed with either compound on its own. LEN and ISL maintained their independent pathways for the development of resistance, and no unexpected RAMs emerged in combination experiments beyond what had been observed previously. When WT virus was used, mutations at RT M184 were the only ISL RAMs observed; M184I alone or as a mixture with wild-type M184 or M184V was observed ubiquitously in all wells with breakthrough at ISL concentrations at or greater than its IC_50_, whether LEN was present or not. When the M184I variant was used to initiate the resistance selection experiments, no additional ISL RAMs were detected, and M184I was maintained in all wells, suggesting that the presence of M184I virus does not rapidly lead to the selection of additional ISL RAMs.

In LEN-only selection experiments, even though viral breakthrough was observed in some wells with LEN concentrations as high as 64-fold over the LEN IC_50_, most of the wells with <8-fold the LEN IC_50_ did not have any changes in CA. The most common change in CA that was observed in the experiments initiated with WT virus was H87Q, which we hypothesized was likely a polymorphism present in the WT virus stock, because (i) it was present in selections with ISL or LEN alone or in combination; (ii) it was observed as a mixture with WT H87 in all but a single well; (iii) it was only present in experiments initiated with WT virus; and (iv) it was a common polymorphism in CA sequences cataloged in the Stanford University HIV Drug Resistance Database (20). Furthermore, antiviral activity assays demonstrated that H87Q resulted in minimal reductions in susceptibility to ISL and LEN when present alone (2.6- and 1.5-fold, respectively) or in combination with other mutations encoding changes in CA (i.e., CA A14T/H87Q had reduced susceptibility of 1.6- and 1.4-fold for ISL and LEN, respectively), confirming the likelihood that H87Q is a CA polymorphism and not associated with resistance.

Outside of CA H87Q, there were CA changes in five wells in the LEN-only resistance selection experiment initiated with WT virus (T54TN/N74D in one well and M66I in two wells at 32-fold of the LEN IC_50_, A14AT and Q67H in one well each at 16-fold of the LEN IC_50_) and in seven wells in the experiment initiated with RT M184I virus (M66I in one well at 64-fold and one well at 32-fold of the LEN IC_50_, N74D or N74ND in five wells at 16- to 32-fold of the LEN IC_50_). M66I, Q67H, and N74D have previously been described, while T54N and A14T are novel. Antiviral assays of CA T54N or CA A14T did not reveal any alterations in susceptibility that was >2-fold. However, the combination of T54N with N74D had an unexpected decrease in susceptibility to LEN (97.5-fold) that would not have been predicted by the results from T54N (1.8-fold) or N74D (7.4-fold) alone. We evaluated the replicative ratio for the T54N, N74D, and T54N/N74D variants using the data from the multiple-cycle antiviral assay. While T54N had a replicative ratio similar to WT, the replicative ratio of N74D and T54N/N74D were reduced by about 50% compared to WT.

**Fig 6:**
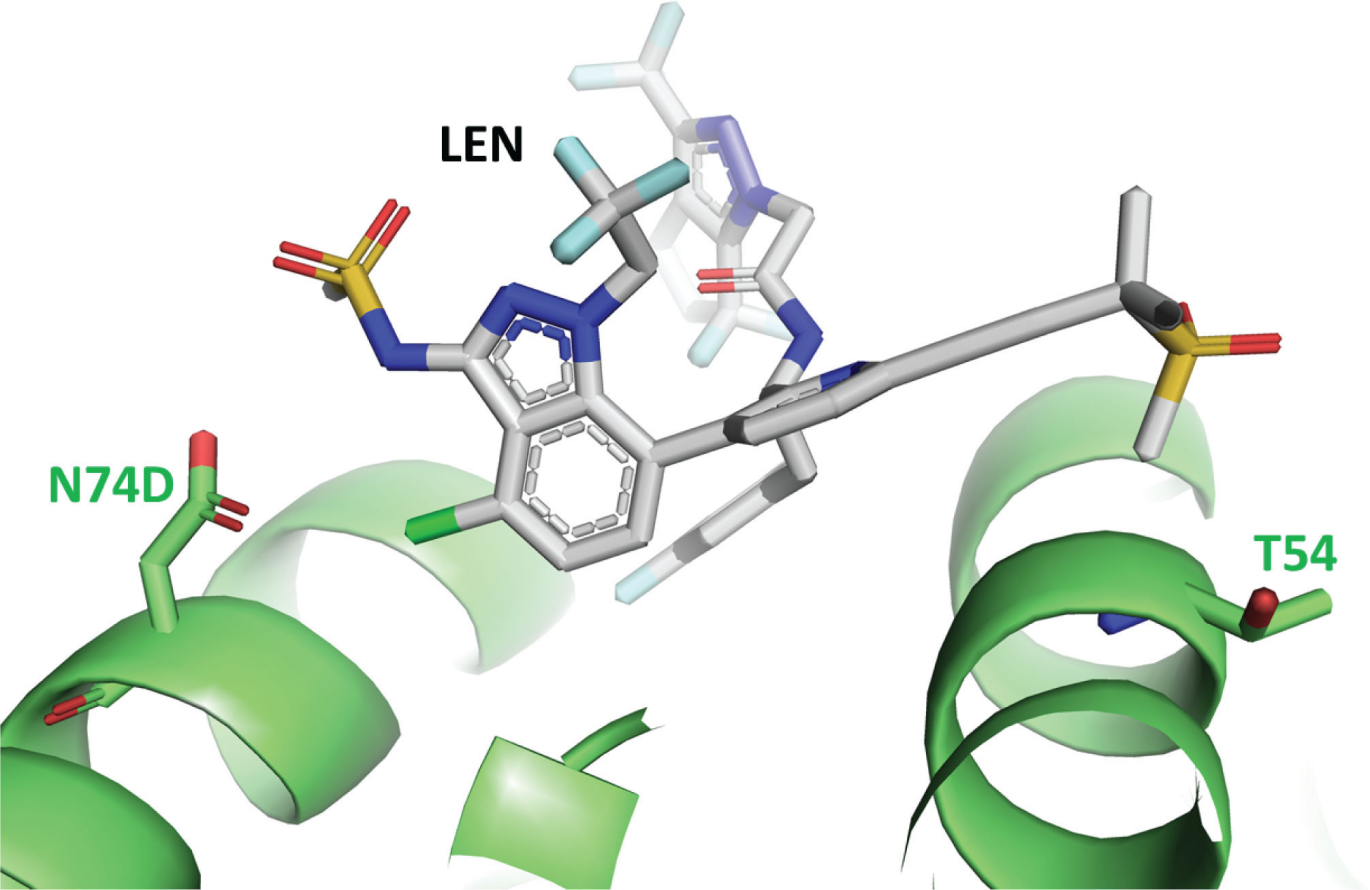
Crystal structure of LEN (gray) bound to CA (N74D) (green), PDB 7RJ4. View of the LEN-binding site in CA. The side chains of D74 and T54 are shown located at opposite ends of the binding pocket for LEN.

In crystal structures of LEN bound to CA (5, 21) and CA with the N74D mutation (22), T54 and N74/D74 are both located in the LEN-binding site and separated by a distance of ~20 Å (Fig. 6). T54 is within 4 Å of the methylsulfonyl group of LEN, whereas N74/D74 is located within 4 Å from the chloro and sulfonamide groups of LEN. N74D has been previously described to result in loss of a hydrogen bond and induced electrostatic repulsion between CA and LEN (22) that causes LEN to move slightly closer to T54 in the pocket. Although a crystal structure of CA with the T54N mutation has not been reported, we hypothesize that the conformational shift of LEN bound to CA containing N74D is less tolerated when T54 is mutated to the larger asparagine side chain, thus accounting for the unexpected decrease in susceptibility to LEN in the combined T54N/N74D mutant virus. In addition, out of ~8,700 CA sequences from individuals in the Stanford Drug Resistance Database (20), there were only three occurrences of T54N and none in combination with N74D. Therefore, the clinical relevance of T54N as an accessory mutation in CA is unknown.

The key observations from the selection experiments are that the combination of ISL with LEN not only resulted in reduced viral breakthrough at higher multiples of each compound but also did not lead to the emergence of LEN RAMs. Altogether, the *in vitro* studies conducted here demonstrating additive antiviral activity, a lack of antagonism or cross-resistance, and a high barrier to the emergence of resistance suggest that ISL and LEN can make an effective two-drug combination for the treatment of HIV-1.

## Data Availability

Sequences from resistance selection experiments have been deposited in GenBank under accession numbers PP827803–PP828572.

## SUPPLEMENTAL MATERIAL

Supplemental tables aac.00334-24-s0001.docx Tables S1 to S3.

ASM does not own the copyrights to Supplemental Material that may be linked to, or accessed through, an article. The authors have granted ASM a non-exclusive, world-wide license to publish the Supplemental Material files. Please contact the corresponding author directly for reuse.
