# Supplementary material for: No antagonism or cross-resistance and a high barrier to the emergence of resistance in vitro for the combination of islatravir and lenacapavir
Source: Antimicrob Agents Chemother. 2024 Jun 12;68(7):e00334-24. doi: 10.1128/aac.00334-24 (PMC11232396; doi:10.1128/aac.00334-24)
Supplement: Supplemental tables — Tables S1 to S3. [file aac.00334-24-s0001.docx]

**Table S1: Plate Layout for Static Resistance Selection Experiments.**

IC_50_ multiple included for each compound is depicted on each well in a 96-well plate with 8 replicates in rows A-H.

|  | **1** | **2** | **3** | **4** | **5** | **6** | **7** | **8** | **9** | **10** | **11** | **12** |
| --- | --- | --- | --- | --- | --- | --- | --- | --- | --- | --- | --- | --- |
| **A** | 128-fold | 64-fold | 32-fold | 16-fold | 8-fold | 4-fold | 2-fold | 1-fold | 0.5-fold | 0.25-fold | 0.125-fold | No compound |
| **B** | 128-fold | 64-fold | 32-fold | 16-fold | 8-fold | 4-fold | 2-fold | 1-fold | 0.5-fold | 0.25-fold | 0.125-fold | No compound |
| **C** | 128-fold | 64-fold | 32-fold | 16-fold | 8-fold | 4-fold | 2-fold | 1-fold | 0.5-fold | 0.25-fold | 0.125-fold | No compound |
| **D** | 128-fold | 64-fold | 32-fold | 16-fold | 8-fold | 4-fold | 2-fold | 1-fold | 0.5-fold | 0.25-fold | 0.125-fold | No compound |
| **E** | 128-fold | 64-fold | 32-fold | 16-fold | 8-fold | 4-fold | 2-fold | 1-fold | 0.5-fold | 0.25-fold | 0.125-fold | No compound |
| **F** | 128-fold | 64-fold | 32-fold | 16-fold | 8-fold | 4-fold | 2-fold | 1-fold | 0.5-fold | 0.25-fold | 0.125-fold | No compound |
| **G** | 128-fold | 64-fold | 32-fold | 16-fold | 8-fold | 4-fold | 2-fold | 1-fold | 0.5-fold | 0.25-fold | 0.125-fold | No compound |
| **H** | 128-fold | 64-fold | 32-fold | 16-fold | 8-fold | 4-fold | 2-fold | 1-fold | 0.5-fold | 0.25-fold | 0.125-fold | No compound |

## **Table S2: ISL and LEN Susceptibility in the Multiple-Cycle Antiviral Assay (100% NHS) for a Panel of HIV-1 Variants Containing LEN RAMs**

| **HIV-1 Variant** | **ISL** | | **LEN** | |
| --- | --- | --- | --- | --- |
|  | **IC_50_ (nM)^a^** | **FC^b^** | **IC_50_ (nM)^a^** | **FC^b^** |
| WT | 1.06 ± 0.21 (n=40)^c^ | 1.0 | 2.39± 0.54 (n=29) | 1.0 |
| CA L56I | 0.87 ± 0.33 (n=3) | 0.8 | 79.46 ± 13.32 (n=8) | 33.2 |
| CA M66I | 0.96 (n=2) | 0.9 | >4202 (n=4) | >1758 |
| CA Q67H | 0.79 ± 0.16 (n=4) | 0.7 | 7.82 ± 0.75 (n=4) | 3.3 |
| CA K70N | 0.77 ± 0.07 (n=4) | 0.7 | 74.94 ± 11.08 (n=8)^f^ | 31.4 |
| CA N74D | 0.78 ± 0.12 (n=4) | 0.7 | 17.64 ± 0.69 (n=4) | 7.4 |
| CA T107N | 0.80 ± 0.21 (n=4) | 0.8 | 5.71 ± 0.79 (n=4) | 2.4 |
| CA Q67H/N74D | 0.78 ± 0.13 (n=6) | 0.7 | 625.42 ± 173.65 (n=7) | 261.7 |
| CA Q67H/N74S | 0.65 ± 0.03 (n=3)^e^ | 0.6 | 14.10 ± 2.74 (n=4) | 5.9 |
| CA Q67H/T107N | 0.89 ± 0.10 (n=4) | 0.8 | 72.59 ± 3.81 (n=4) | 30.4 |
| RT M184V | 7.25 ± 1.81 (n=18) | 6.8 | 0.98 ± 0.26 (n=4) | 0.4 |
| CA M66I/RT M184V | 6.02 ± 1.47 (n=5) | 5.7 | >2192 (n=7)^d^ | >917 |
| CA Q67H/RT M184V | 6.00 ± 0.86 (n=4) | 5.7 | 9.36 ± 1.46 (n=4) | 3.9 |
| CA N74D/RT M184V | 5.05 ± 0.45 (n=3) | 4.8 | 18.32 ± 0.58 (n=3) | 7.7 |
| CA=capsid; FC=fold change; HIV-1=human immunodeficiency virus type 1; IC_50_= Half maximal inhibitory concentration; ISL=islatravir; LEN=lenacapavir; RT=reverse transcriptase WT=wild-type.   1. IC_50_ is displayed as geometric mean ± standard deviation. 2. FC is calculated as (IC_50_ against mutant isolate) / (IC_50_ against WT) for each test article. 3. LEN IC_50_ with CA M66I/RT M184V virus is the geometric mean of 7 repeats. Four were within the range of the assay and 3 were >4202 nM. Values of >4202 were amended to 4202 in order to calculate the geometric mean. | | | | |

## **Table S3: ISL and LEN Susceptibility in the Multiple-Cycle Antiviral Assay (100% NHS) for a Panel of HIV-1 Variants Observed in ISL/LEN Static Resistance Selection Experiments**

| **HIV-1 Variant** | **ISL** | | **LEN** | |
| --- | --- | --- | --- | --- |
|  | **IC_50_ (nM)^a^** | **FC^b^** | **IC_50_ (nM)^a^** | **FC^b^** |
| WT | 1.06 ± 0.21 (n=40) | 1.0 | 2.39± 0.54 (n=29) | 1.0 |
| CA A14T | 1.31 ± 0.06 (n=3) | 1.2 | 2.92 ± 0.27 (n=4) | 1.2 |
| CA T54N | 0.77 ± 0.10 (n=3) | 0.7 | 4.21 ± 0.67 (n=4) | 1.8 |
| CA H87Q | 2.77 ± 0.10 (n=3) | 2.6 | 3.70 ± 0.16 (n=4) | 1.5 |
| CA A14T/H87Q | 1.67 ± 0.14 (n=3) | 1.6 | 3.32 ± 0.58 (n=4) | 1.4 |
| CA T54N/N74D | 1.60 (n=2) | 1.5 | 232.93 ± 34.21 (n=3) | 97.5 |
| CA Q67H/H87Q | 2.38 ± 0.53 (n=3) | 2.2 | 20.84 ± 2.15 (n=3) | 8.7 |
| CA M66I/RT M184I | 10.87 ± 1.30 (n=3) | 10.3 | 937.62 ± 416.93 (n=3) | 392.3 |
| CA N74D/RT M184I | 6.71 ± 0.99 (n=3)^e^ | 6.3 | 25.64 ± 6.29 (n=4) | 10.7 |
| CA H87Q/RT M184I | 14.34 ± 1.81 (n=7) | 13.5 | 2.84 ± 0.45 (n=8) | 1.2 |
| CA H87Q/RT M184V | 18.10 ± 2.09 (n=8) | 17.1 | 3.12 ± 0.35 (n=7) | 1.3 |
| CA=capsid; FC=fold change; HIV-1=human immunodeficiency virus type 1; IC_50_= Half maximal inhibitory concentration; ISL=islatravir; LEN=lenacapavir; RT=reverse transcriptase WT=wild-type.  a IC_50_ is displayed as geometric mean ± standard deviation.  b FC is calculated as (IC_50_ against mutant isolate) / (IC_50_ against WT) for each test article. | | | | |
